# Relationship between radiation pneumonitis and organizing pneumonia after radiotherapy for breast cancer

**DOI:** 10.1186/1748-717X-8-56

**Published:** 2013-03-08

**Authors:** Yumi Oie, Yasunori Saito, Masanao Kato, Fumitaka Ito, Hidekazu Hattori, Hiroshi Toyama, Hidetoshi Kobayashi, Kazuhiro Katada

**Affiliations:** 1 Department of Radiology, Fujita Health University School of Medicine, 1-98 Dengakugakubo, Kutsukake, Toyoake, Aichi 470-1192, Japan; 2 Division of Radiology, Fujita Health University Hospital, Toyoake, Japan

**Keywords:** Organizing pneumonia (OP), Breast cancer, Radiation pneumonitis (RP)

## Abstract

**Background:**

Radiation pneumonitis (RP) and organizing pneumonia (OP) are the two main types of lung damage that can occur after lung irradiation. The goal of this study was to evaluate the relationship between RP and OP after irradiation for breast cancer.

**Methods:**

Four hundred and twenty-eight patients who underwent radiotherapy for breast cancer were identified. The whole breast was irradiated with two tangential photon beams. Chest computed tomography (CT) scan were performed when patients showed any symptoms that were suspicious for pneumonitis.

**Results:**

Five patients (1.2%) were diagnosed with OP. All five patients showed ground glass opacities and consolidation of the border of the lesion of RP in the radiation fields. Infiltration of OP spread from the site of RP to the hilum of the ipsilateral lung. Between RP and OP, a free region space (FRS) could be detected.

**Conclusions:**

OP is closely related to RP. All OP lesions developed near the site of RP.

## Background

Postoperative radiotherapy plays an important role in the management of breast cancer and can reduce the local and regional recurrence, thereby improving outcomes [1,2]. One study reported that the overall frequency of Grade 2 or 3 adverse events after radiotherapy was 3.8% in Japanese women with breast cancer [3]. Adverse events following irradiation for breast cancer include pain and pigmentation of the chest wall, skin ulcerations, soft tissue fibrosis, rib fractures, myocardial infarction, pericardial effusion, and symptomatic or asymptomatic lung damage. Pneumonitis is one type of radiation-induced lung damage, and it can be divided into two types: radiation pneumonitis (RP) and organizing pneumonia (OP) [4]. OP is included RP in the broad sense, but OP in this paper is defined OP without RP.

RP is a form of acute or subacute lung damage related to the dose of radiation. It develops along the irradiated fields and results in pulmonary fibrosis. Asymptomatic RP is commonly observed, while symptomatic RP is rare. Past study reported majority (74%) of patients with RP were asymptomatic [5]. OP is a form of subacute lung damage that occurs independent of the radiation dose. It is identified inside and outside of the tangential irradiated field and does not result in pulmonary fibrosis. Although OP is rare, when it does occur, it is usually found in symptomatic. RP and OP are suspected to belong in another etiological category. The goal of the present study is to investigate the relationship between RP and OP.

## Methods

Between January 2002 and December 2009, 428 patients with breast cancer underwent radiotherapy at Fujita Health University in Japan. Patients undergoing breast-conserving surgery, total mastectomy, or no surgery were included. Patients with recurrent breast cancer and patients who were lost to follow-up within 1 year were excluded. One patient with bilateral breast cancer treated with radiotherapy at separate times was counted as two cases. Six patients with bilateral breast cancer who were treated with radiotherapy to both breasts simultaneously were counted as six patients and 12 breasts. Thus, the total number of patients treated with irradiation was 428, and the total number of irradiated breasts was 434.

Patients were asked to follow-up at the hospital after radiotherapy. When respiratory symptoms and/or general symptoms appeared, computed tomography (CT) was performed. The diagnosis of OP was based on the criteria proposed by Crestani et al. [4]: (1) radiation to the breast within 12 months, (2) general and/or respiratory symptoms lasting for 2 weeks; (3) radiographic lung infiltration outside the radiation port; and (4) no evidence of specific cause.

Characteristics of the 428 patients are shown Table 1. All patients were women. The median age was 56 years. Collagen vascular disease was found in four patients, including two patients with rheumatoid arthritis, one patient with systemic lupus erythematosus (SLE), and one patient with polymyositis. Allergic disease was found in 20 patients, including 15 patients with asthma, and five patients with atopic dermatitis. Lung disease was found in two patients, including one patient with interstitial pneumonia and another patient with tuberculosis. The details of radiotherapy are shown in Table 2. Whole breast irradiation and chest wall irradiation with two tangential 4 MV photon beams was performed. For irradiation of the regional lymph nodes, an anterior photon beam was used. To boost irradiation to the tumor bed, an electron beam was used. The details of the adjuvant therapy are shown in Table 3. No patient was treated with concurrent chemoradiation therapy.

**Table 1:** Patient characteristics (n = 428)

Age (y)	
20 ~ 29	3
30 ~ 39	35
40 ~ 49	104
50 ~ 59	117
60 ~ 69	124
70 ~ 79	41
80 ~ 89	4
Irradiated breast (right/left/bilateral)	211/208/9
Collagen vascular disease (yes/no)	4/424
Allergy disease (yes/no)	20/408
Lung disease (yes/no)	2/426
Diabetes (yes/no)	29/399
Smoking habit (yes/no/unknown)	30/371/27
Clinical stage (UICC)	
0	30
I	215
IIA	110
IIB	47
IIIA	11
IIIB	17
IIIC	3
IV	1

**Table 2:** Radiation therapy details

Whole-breast irradiation / Chest wall irradiation	400/34
50 Gy in 25 fractions	433
54 Gy in 27 fractions	1
Irradiation to the regional lymph nodes (yes/no)	45/388
supraclavicular region of affected side	
50 Gy in 25 fractions	26
40 Gy in 20 fractions	5
supraclavicular and parasternal region of affected side	
50 Gy in 25 fractions	4
45 Gy in 23 fractions	1
40 Gy in 20 fractions	8
supraclavicular and parasternal region of bilateral side	
40 Gy in 20 fractions	1
Boost to tumor bed (yes/no)	31/403
9 Gy in 3 fractions	30
6 Gy in 3 fractions	1

**Table 3:** Adjuvant therapy details

Chemotherapy (yes/no)	181/247
FEC-T	82
AC-T	33
FEC	20
AC	9
EC-T	8
CMF	7
TC	5
Docetaxel	5
Others	12
Concurrent endocrine therapy	
Tamoxifen or Tremifen (yes/no)	157/271
Aromatase inhibitor(yes/no)	177/251
Trastuzumab(yes/no)	27/401

FEC-T = 5-fluorouracil, epirubicin, cyclophosphamide plus taxane.

AC-T = adriamycin, cyclophosphamide plus taxane.

FEC = 5-fluorouracil, epirubicin, cyclophosphamide.

AC = adriamycin, cyclophosphamide.

EC-T = epirubicin, cyclophosphamide plus taxane.

CMF = cyclophosphamide, methotrexate, 5-fluorouracil.

TC = docetaxel, cyclophosphamide.

## Results

Five (1.2%) of the 428 patients were diagnosed with OP (Table 4). All patients were diagnosed by clinical findings and imaging findings. Transbronchial lung biopsy (TBLB) was performed for the diagnosis in three cases. OP was revealed in two patients. In one patient, only non-specific change was revealed by TBLB specimens. Infection was ruled out in all five patients. All five patients were nonsmokers, and none of these five patients had a history of allergic disease, collagen vascular disease, lung disease, or diabetes. Ages of these five patients were 52, 54, 55, 66, 68 years (median, 55 years old). Four of the five patients had right breast cancer, and one had bilateral breast cancer. Thus, there were six affected breasts in total. Clinical staging by the tumor-node-metastasis classification system showed that four breasts were T1N0M0 and that one breast was T2N0M0. All five patients had undergone breast-conserving surgery. For whole-breast irradiation, all breasts received 50 Gy in 25 fractions. The photon energy was 4 MV in all five patients. None of the five patients underwent irradiation to the regional lymph nodes (including the supraclavicular region, parasternal region or axillary region) or boost irradiation to the tumor bed. The percent of V_20Gy_ compared with whole lung volume of each five patients was under 15%. Three (60%) of the five patients received chemotherapy before radiotherapy. Two (40%) of the five patients received concurrent endocrine therapy (tamoxifen and aromatase inhibitor, respectively). No patients received trastuzumab.

**Table 4:** Clinical characteristics of five patients with OP

**Patient no.**	**Age (y)**	**Irradiated breast**	**Drug before RT**	**Drug concurrent with RT**	**Period before onset, after RT (days)**	**Initial area of pulmonary findings**	**Frequency of relapse**	**Relapse in contralateral area**	**Duration of steroid administration**
1	66	Right	FEC	―	229	Right upper• middle• lower lobe	once	No	385
2	52	Right	FEC	Tamoxifen	218	Right middle lobe	once	No	206
3	68	Right	―	―	229	Right middle lobe	5 times	Yes	799
4	54	Right	―	Anastrozole	168	Right middle lobe	once	Yes	244
5	55	Bilateral	CMF	Exemestane	170	Bilateral middle lobe	―	No	38
median	55				218				244

### Clinical course of patients with OP

The interval from completion of radiotherapy to occurrence of symptoms ranged from 170 to 229 days (median, 218 days). The initial clinical symptoms in the five patients were cough (four patients), fever (three patients), chest pain (two patients), and general malaise (one patient). In all five patients, the initial abnormal pulmonary findings were observed in the ipsilateral area, including the irradiated field. All patients were given steroids after being diagnosed with OP. The duration of steroid administration ranged from 38 days to 799 days (median, 244 days). Clinical and physiologic improvement and normalization of the pulmonary opacities occurred rapidly in all five patients after steroid administration, but relapse occurred in four patients during the steroid taper (two patients) or soon after steroid withdrawal (two patients). Five episodes of relapse occurred in one patient. One episode of relapse occurred in three patients. Migration of consolidation and of ground-glass opacities (GGO) was confirmed on CT examination in all five patients. Abnormal pulmonary findings appeared on the contralateral side in two of five patients at the relapse time.

### CT findings

**Figure 1:**
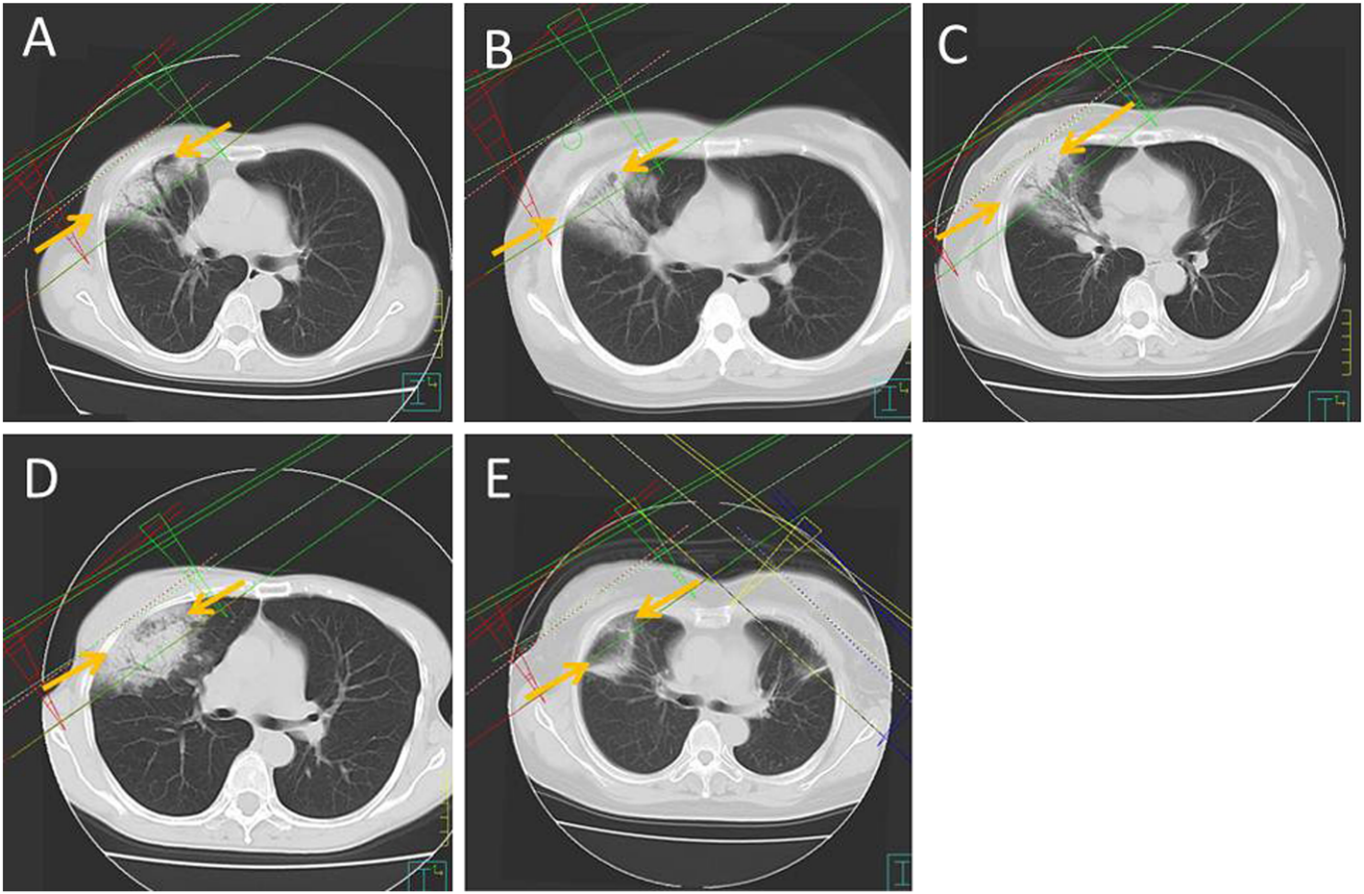
**Fusion of CT images at the time of diagnosis of OP and treatment planning images (A = patient 1, B = patient 2, C = patient 3, D = patient 4, E = patient 5).** There were free regions between the radiation lesions and the radiation-induced OP (arrow).

**Figure 2:**
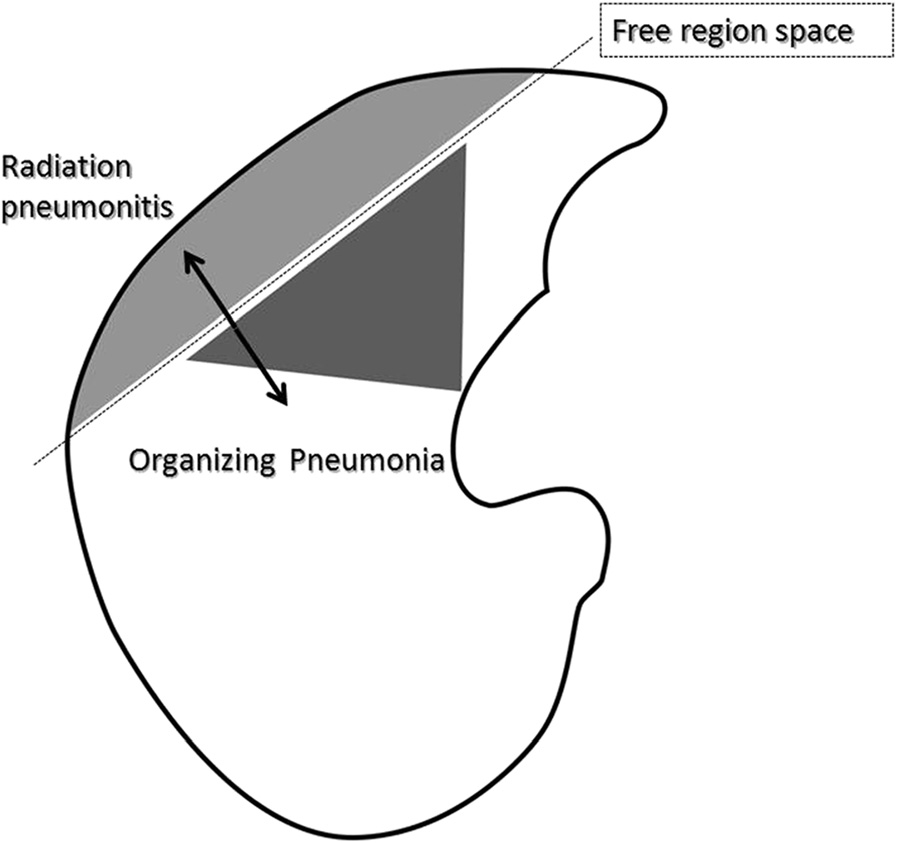
Close-up schema of the relationship between RP lesion and OP lesion.

All five patients showed GGO and consolidation of RP in the radiation fields. Initial OP lesions were in close proximity to the RP lesions, but the lesions were not connected. Infiltration of OP spread from the site of RP to the hilum of the ipsilateral lung. Between both lesions, there was a free region space (FRS) (Figures 1, 2). FRS between the lesions of RP and OP were recognized in all five patients.

## Discussion

Radiation-induced lung injury can be divided into RP and OP [4]. RP is different from OP in several ways: (1) RP occurs during radiotherapy or shortly after the completion of radiotherapy, while OP occurs within 1 year after completion of radiotherapy; (2) RP lesions are characterized by alveolar opacities and are limited to the irradiated area, while OP lesions are characterized by lung infiltrates outside the radiation field and frequently migrate; (3) RP occurs in almost all patients whose lungs have been irradiation (i.e., not only in breast cancer patients), while the incidence of OP in patients with breast cancer or other malignancies is low; (4)RP always results in fibrosis and never relapses, while OP usually resolves without fibrosis but commonly relapses when the glucocorticoid is withdrawn. Further, OP lesions tend to migrate (i.e., the relapsed lesions often occur in another area, or even opposite side, relative to the initial lesions).

Imaging studies suggest that RP and OP may be related to one another. On CT scan, RP lesions occur close to the thoracic wall, and most OP lesions develop in close proximity to the RP lesions. RP occurs at site that have been irradiated with a dose > 20 Gy. Ogo et al. reported most OP lesions develop outside of the irradiated field and few cases (2 of 37) show no relation with RP [6]. OP shadows extend beyond the radiation port and often migrate to another side of the lung when OP relapsed. Although OP has been suggested to be an indirect effect of radiation, radiotherapy may play an important role in the development of OP [4]. OP is a form of lung toxicity that likely arises between some interaction between radiotherapy and the immune system [6], which may also explain the occurrence of OP lesions outside the irradiated field.

It is an important question why OP occurs after radiotherapy for breast cancer more frequently than after radiotherapy for other malignancies. Lungs are often irradiated for the treatment for malignancies. Late damage to the lung, which usually manifests as fibrosis, is a radiation dose-dependent occurrence in patients undergoing radiotherapy for lung cancer, esophageal cancer and mediastinal tumors. The incidence of OP after radiotherapy in patients with breast cancer is 1.8-2.9% [6-11]. In contrast, RP occurs much more commonly after radiotherapy in patients with lung cancer [12-14] or esophagus cancer [15,16]. Several reports describe the occurrence of OP after radiotherapy in patients with lung cancer [17-19].

The reasons why the incidence of OP is lower after radiotherapy for lung cancer, esophageal cancer, and mediastinal tumor than after radiotherapy for breast cancer remain unknown. One explanation could be related to the fact that the dose distribution of radiotherapy for breast cancers differs from that for other malignancies. Further, tangential irradiation is used to treat breast cancer but not other malignancies.

The relationship between the irradiated field and RP and OP is illustrated in Figure 2. The inner lines composed by the opposed tangential fields form a straight line, which indicates a sharp decline in absorbed dose. Tangential irradiation can reduce the volume of the irradiated lung when compared with other irradiation methods, such as opposing portal irradiation and single field irradiation.

**Figure 3:**
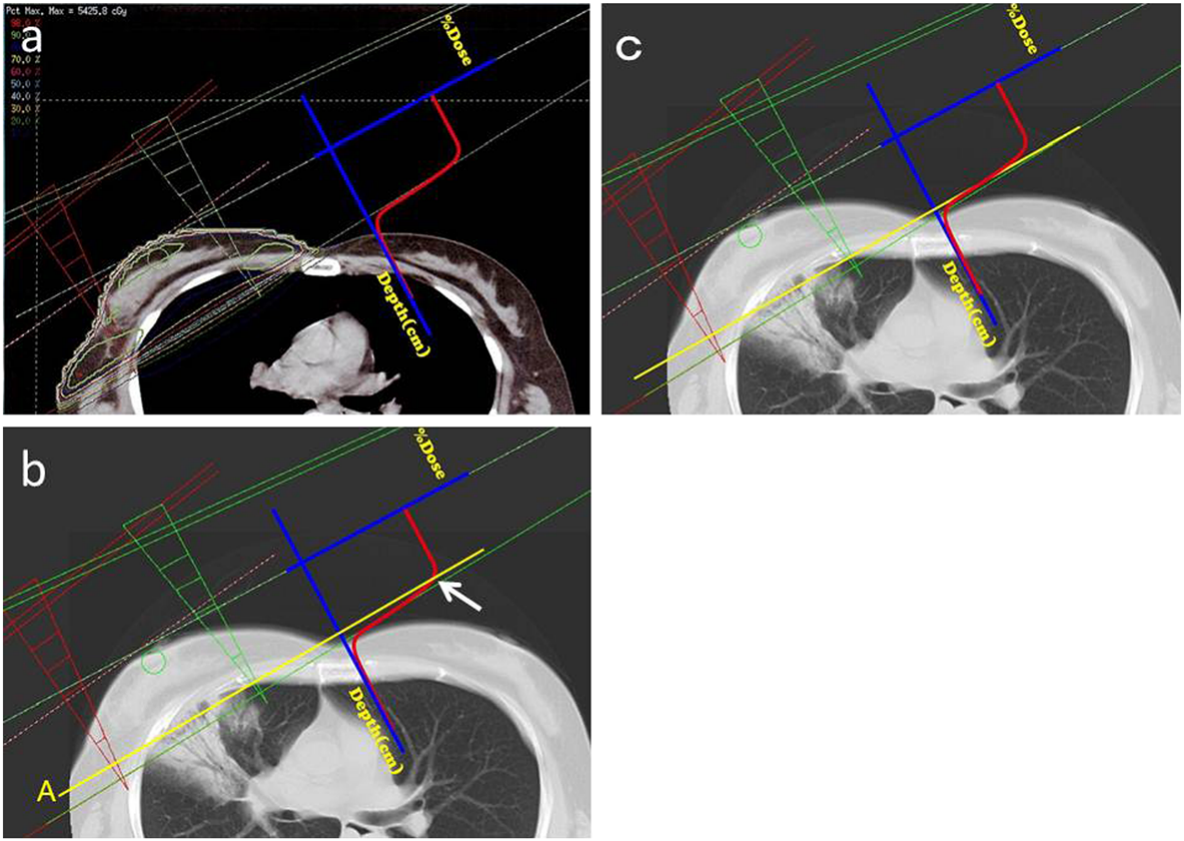
**Fusion of CT images and %dose-depth curve. a**. CT image at the radiation treatment planning and %dose-depth curve. % Dose decreased sharply at the tangential line. **b**. CT imaging at the diagnosis of OP and %dose-depth curve. The line (A) shows the border between RP and FRS. The intersection (arrow) of the line with the %depth-dose curve is associated with a radiation dose > 20 Gy. The RP lesion might appear closer to the chest wall because of lung volume reduction related to fibrotic change following RP. **c**. The 20 Gy point of the %dose-depth curve moved to the border of RP. The FRS corresponds with the region of steep change of distribution.

%Dose-depth curves are shown relative to dose distributions in Figure 3a. The tolerance dose of RP is about 20 Gy; the more V_20Gy_ increases, the higher the incidence of RP [12,20-22].

The border of the RP is closer to the thoracic wall than the point of 20 Gy on the planning CT (Figure 3b). This shift of the RP might actually be related to a decrease in lung volume because of fibrous changes after RP. The dose distribution should be shifted to coincide with the point of 20 Gy with the border of RP (Figure 3). This shifted dose distribution is suspected to be the actual dose distribution on the CT at the onset of OP.

RP and OP are closely related to one another. However, between the lesions of RP and OP, FRS was detected on the CT images. The same findings were seen in a previous report [6]. Fusion of CT images at the onset of OP and the planned dose distributions of radiotherapy is shown in Figure 3. FRS on the CT images was located at the region next to the rapidly decreasing dose area. The irradiated dose of FRS is suspected to occur at < 20 Gy, but not at zero. This dose area from 0 to 20Gy may allow an immunologic reaction that results in development of the initial OP point (or area).

The present study suggests that the OP lesions are closely connected to the FRS detected on the CT images, which arise next to the rapidly decreasing dose area. In conformal irradiation and intensity modulated radiation therapy (IMRT), the dose contribution sometimes shows a steep dose gradient. Further pathology studies are needed to investigate this hypothesis.

## Conclusions

OP occurred in 1.2% of patients with breast cancer who underwent radiotherapy. Most OP develops after irradiation for breast cancer. A low dose region and a steep dose gradient may be associated with the onset of the OP lesion. This finding suggests that frequent and active follow-up CT after radiotherapy may be needed.

## Abbreviations

OP: Organizing pneumonia; RP: Radiation pneumonitis; TBLB: Transbronchial lung biopsy; GGO: Ground glass opacity; V20Gy: Lung volume covered with 20 Gy or more; FRS: Free region space; FI: Fusion image; IMRT: Intensity modulated radiation therapy.

## Competing interests

The authors declare that they have no competing interests.

## Authors’ contributions

YO: contributed to the design of the search strategy, data abstraction, data analysis, data interpretation, the drafting of the manuscript, and critical revision of the article for important intellectual content. FI: contributed to the design of the search strategy. HH: contributed to the design of the search strategy. HT: contributed to the design of the search strategy and critical revision of the article for important intellectual content. HK: contributed to the design of the search strategy, data analysis, and critical revision of the article for important intellectual content. KK: contributed to the design of the search strategy and critical revision of the article for important intellectual content. YS: contributed to the drafting of the manuscript. MS: contributed to the drafting of the manuscript. All authors read and approved the final manuscript.
